# Emerging trends in peer review—a survey

**DOI:** 10.3389/fnins.2015.00169

**Published:** 2015-05-27

**Authors:** Richard Walker, Pascal Rocha da Silva

**Affiliations:** ^1^Blue Brain Project, École polytechnique fédérale de Lausanne ENT Center for Brain Simulation Blue Brain Project / Human Brain ProjectGeneva, Switzerland; ^2^Frontiers, École polytechnique fédérale de Lausanne Innovation ParkLausanne, Switzerland

**Keywords:** peer review, preprint servers, open peer review, non-selective review, anonymity, open access, interactive review, impact metrics

## Abstract

“Classical peer review” has been subject to intense criticism for slowing down the publication process, bias against specific categories of paper and author, unreliability, inability to detect errors and fraud, unethical practices, and the lack of recognition for unpaid reviewers. This paper surveys innovative forms of peer review that attempt to address these issues. Based on an initial literature review, we construct a sample of 82 channels of scientific communication covering all forms of review identified by the survey, and analyze the review mechanisms used by each channel. We identify two major trends: the rapidly expanding role of preprint servers (e.g., *ArXiv*) that dispense with traditional peer review altogether, and the growth of “non-selective review,” focusing on papers' scientific quality rather than their perceived importance and novelty. Other potentially important developments include forms of “open review,” which remove reviewer anonymity, and interactive review, as well as new mechanisms for post-publication review and out-of-channel reader commentary, especially critical commentary targeting high profile papers. One of the strongest findings of the survey is the persistence of major differences between the peer review processes used by different disciplines. None of these differences is likely to disappear in the foreseeable future. The most likely scenario for the coming years is thus continued diversification, in which different review mechanisms serve different author, reader, and publisher needs. Relatively little is known about the impact of these innovations on the problems they address. These are important questions for future quantitative research.

## Introduction

**Figure 1:**
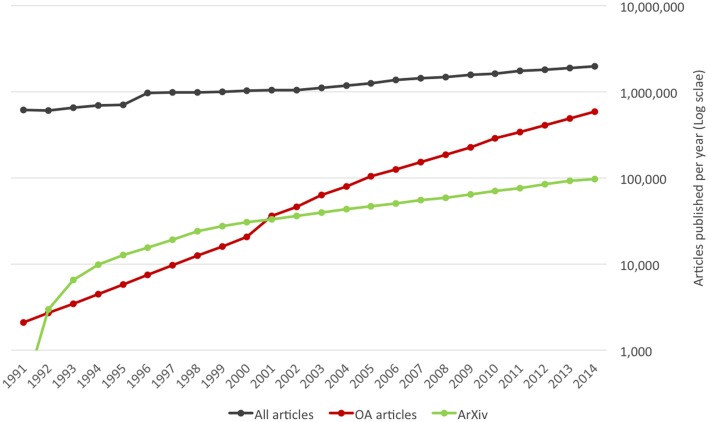
**All articles: annual production of original research and review articles indexed by Scopus (data retrieved February 13, 2015)**. OA articles: annual number of original research and review articles in Open Access Journals for period 2000–2011 from Laakso and Björk (2012); data before 2000 and after 2011 estimated from known growth rates. ArXiv: data calculated from monthly submission data in (ArXiv, 2015a).

**Figure 2:**
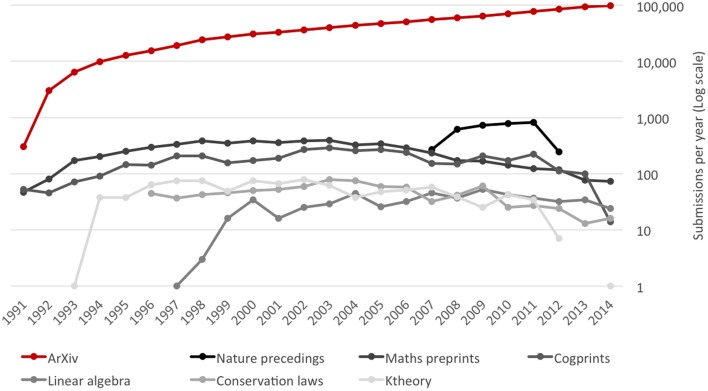
**Trends in submissions to selected preprint servers (1991–2014)**. ArXiv: data calculated from monthly submission data in ArXiv (2015a). Nature Precedings: data calculated using advanced search function in Nature Precedings (2015). Maths preprints: data calculated from year by year lists of papers at Mathematics on the Web (2015). Cogprints: data from Cogprints (2015). Linear Algebra: data calculated from complete list of papers at Linear Algebraic Groups and Related Structures Preprint Server (2015). Conservation laws: data calculated from year by year lists of papers in Preprints on Conservation Laws (2015). Ktheory: data calculated from complete list of papers at K-theory Preprint Archives (2015).

Web technologies, rapid reductions in the cost of computer storage and network communications, and the advent of specialized search engines have revolutionized the economics of scientific publishing, initiating disruptive changes that are still in progress. Perhaps the most obvious has been the enormous increase in the number of papers published, which has grown at an average rate of 5% per annum, and shows no sign of slowing down. Equally important has been the emergence of Open Access publishing (see Figure 1) and preprint servers (see Figure 2) as new modes of academic publishing, together with new search engines that have made it possible for readers to search and navigate an ever-growing literature.

But another set of changes has attracted less attention. Until the early 1990s, nearly all papers were evaluated using “classical peer review” (see below). Today, by contrast, new review processes are emerging—in most cases driven by Open Access Publishing. While some of these innovations are highly experimental and several have been unsuccessful, others are already widespread. In particular, two key innovations (shown in the timeline in Table 1) have had a major impact on scientific publishing.

**Table 1:** **Major innovations in peer review—a timeline**.

1732	*Royal Society of Edinburgh* uses peer review for the first time
1893	*British Medical Journal* adopts peer review
1959	*Current Anthropology* introduces Open Peer Commentary
1964	*Nature* introduces peer review
1976	*Lancet* introduces peer review
1978	*Brain and Behavioral Sciences* introduces Open Peer Commentary
1991	Launch of *ArXiv*
1999	*British Medical Journal* begins to reveal reviewer names to authors
2000	*BioMed Central* (BMC) adopts open review for all its medical journals
2001	*Atmospheric Chemistry and Physics* introduces two-stage review process in which papers are published as “discussion papers” before formal review
2003	First article on *BMC Medicine*
2006	First article on *PLOS ONE* using non-selective review
2006	*Nature* experiment in community review
2007	First article in *Frontiers* using non-selective interactive review and including names of editor and reviewers
2007	*Nature* launches commercial preprint server (*Nature Precedings)*
2010	*Shakespeare Quarterly* experiment in open review
2011	*BMJ Group* launches *BMJ Open*
2012	Launch of several new journals adopting open review (*GigaScience, PeerJ, eLife, F1000 research)*
2012	*Nature Precedings* ceases to accept new submissions
2013	*Nature Genetics* and *Nature Climate Change* offer double blind review

The first is the rapid growth of non-commercial pre-print servers, primarily *ArXiv*, which make papers available to readers without any prior review or with only a minimal “access review”—a rapid review to ensure that the paper meets minimum standards for scientific publication and/or to check the credentials of the authors. The second, driven by the Open Access movement, is the emergence of non-selective review processes, which consider only the scientific quality of a paper and not its “importance” and “novelty,” and the introduction of various forms of open and interactive review.

In the 1970s, 1980s, and 1990s, the strengths and weaknesses of classical peer review were the object of a large number of experimental and observational studies. However, the equivalent literature for non-classical forms of review is small, with most of the debate taking place in the opinion sections of journals, and in the “blogosphere.” This paper surveys emerging trends, summarizes the (limited) evidence about their impact, and identifies gaps in our current knowledge that call for future research.

## Classical peer review

For the purposes of this study we define classical peer review as a process which:

- Assesses the suitability of a manuscript for publication, and provides feedback to authors, helping them to improve the quality of their manuscripts
- Follows formal procedures and assessment criteria
- Takes place before publication
- Is highly selective
- Assesses manuscripts in terms of their novelty, “importance for the field” and “interest for a broad readership”
- Is conducted by a small number of editor-selected expert reviewers, whose names are not revealed to authors or readers, and who make their assessments without any direct interaction among themselves or with authors
- Concludes with a publication decision taken by the editor(s).

The history of classical peer review dates back to at least 1732, when the Royal Society of Edinburgh set up a committee to select the papers it would publish in its *Philosophical Transactions* (Spier, 2002). It continued to be used occasionally throughout the 19th and early twentieth century. For instance, the *BMJ* began to systematically review submissions as early as 1893 (Burnham, 1990). However, the practice became general only with the exponential growth in scientific production that began after the end of World War II, and the introduction of the photocopier which made it easy to distribute papers to reviewers (Spier, 2002). One of the pioneers was the *Journal of the American Medical Association (JAMA)*, which began to use peer review in the late 1940s (Spier, 2002). *Nature* introduced it only in 1964. *Lancet* waited until 1976 (Benos et al., 2007).

Despite its relatively recent origins, and despite frequent criticism (see below), there is widespread consensus about its advantages. The anonymity of the review process allows reviewers to express critical views freely, without fear of retaliation from authors. Lack of interaction among reviewers prevents high prestige or forceful reviewers from dominating the review process. Authors benefit from the prestige that comes with publication on peer-reviewed journals. Institutions use peer-reviewed publications as an indicator of scientific productivity and value. For publishers of paper journals, with high marginal production costs and limited page budgets, classical peer review provides an effective mechanism for selecting articles likely to attract a large number of citations, and improving impact factors. Experimental studies and surveys of authors' and reviewers' opinions concur that peer review improves the quality of scientific publications, filtering out low quality work, catching errors, improving the writing and providing readers with a useful signal of quality (Bradley, 1981; MacNealy et al., 1994; Fletcher and Fletcher, 1997; Armstrong, 1998; DeCoursey, 1999; Ware, 2008). Nonetheless, there is also a consensus that current models of peer review are less than ideal.

The key objections can be summarized as follows:

1. **Delay**. Classical peer review represents an unacceptable slowing down of the scientific process (Armstrong, 1997; Benos et al., 2007), creating delays and extra work for authors, while simultaneously preventing readers from accessing results they need for their own research or in the clinic. In some disciplines, the “hassles” associated with the review process are so serious that high profile authors have reduced the number of papers they submit to major journals and increased their use of other, more efficient channels of communication (Ellison, 2011)—some reviewed later in this paper.
2. **Bias against specific categories of paper**. Many seminal papers that later won Nobel prizes for their authors were initially rejected by reviewers (Campanario, 2009). Two experimental studies show that reviewers prefer papers reporting positive results to those reporting negative or mixed results, even when the underlying methodologies are identical (Mahoney, 1977; Emerson et al., 2010). Other commentators have pointed to a systematic bias against replication studies (Kerr et al., 1977; Campanario, 1998).
3. **Social and cognitive biases**. Studies have suggested that classical peer review may be affected by bias against female authors (Lloyd, 1990; Tregenza, 2002; Budden et al., 2008), authors from particular geographical areas (Link, 1998; Bornmann and Daniel, 2009), authors from countries where English is not a native language (Herrera, 1999), and authors from low prestige institutions (Peters and Ceci, 1982). Reviewers have also been shown to use inappropriate cues to identify papers worthy of publication, including the use of sophisticated statistical methodology, even when this was not appropriate (Travis and Collins, 1991). In other studies, reviewers were shown to prefer papers with complex sentences and obscure vocabulary to papers with identical content written in clearer language (Armstrong, 1980, 1997).
4. **Unreliability**. Several authors have argued that reviewers' assessments of the quality of scientific papers are not reliable. An experimental study in which different reviewers were asked to review the same paper, showed extremely poor agreement among referees, both on their overall recommendations and on their responses to individual items on the review questionnaire (Mahoney, 1977). A more recent retrospective study supports this conclusion, showing that reviewers agreed on recommendations at levels little better than chance (Kravitz et al., 2010). Most journals use just 2–3 reviewers. Mathematical modeling suggests that the results of such a process are unlikely to be much better than those from a lottery (Herron, 2012).
5. **Inability to detect errors and fraud**. Classical peer review frequently fails to detect papers containing serious errors in methodology, manipulated figures or even fabricated data (Schroter et al., 2004; Nature, 2006b). High-profile cases in which the system has obviously failed (The Economist, 2014; PubPeer, 2015) include a recently retracted Nature paper (Obokata et al., 2014). Other studies have gone so far as to suggest that the majority of published results in the biomedical sciences are false (Ioannidis, 2005) or impossible to replicate (Prinz et al., 2011), and that many researchers engage in questionable research practices (John et al., 2012). If it is considered that most papers are reviewed by just 2–3 unpaid reviewers, who cannot perform replication experiments, and may not have the expertise to evaluate some aspects of the paper such as statistical methodology (Ozonoff, 2006), these findings are not surprising.
6. **Lack of transparency**—**unethical practices:** Classical peer review is a non-transparent process that puts editors in a position to exert unfair influence over the review process, choosing reviewers, favorable or unfavorable to a particular thesis or a particular author (Travis and Collins, 1991). It also provides ample opportunities for “self-serving reviewer behavior” (Kriegeskorte et al., 2012), as described in frequent anecdotal reports of reviewers who have behaved unethically, rejecting, delaying or copying work by competitors (Sieber, 2012).
7. **Lack of recognition for reviewers**. Anonymous review provides no recognition for reviewers' unpaid work, and their often substantial contributions to the papers they review.

Few critics of classical peer review believe that it should be abolished: one author compared it to democracy: “a system full of problems but the least worst (sic) we have” (Smith, 2006). It is possible, furthermore, that some of the criticisms described above are overstated, or have become less relevant than in the past. For instance, findings of gender bias have been challenged (Gilbert et al., 1994; Webb et al., 2008) and a recent quantitative study by one of the authors found little supporting evidence for this, or for other forms of “social bias” (Walker et al., 2015). Nonetheless, many other problems are widely recognized, inspiring attempts at reform. It is these that we review below.

## Methods

### Definitions

For the purposes of this study, a *paper* or *article* is defined as a document describing, reviewing or discussing results, observations, hypotheses, theories, methods, and other outputs of academic and engineering research. *Publication* is the act whereby an author makes a paper available to readers. *Publication channels* are media (e.g., preprint-servers, print or online journals) that allow the publication of papers, reviews of papers or user commentary on papers (see below). A *publisher* is a commercial or non-commercial organization responsible for the management of one or more publication channels. *Peer review* is a formal process for the assessment of a manuscript. It may be *in channel* (managed by the publication channel where accepted papers will appear), or *out of channel* (offered by *review channels*). *In channel* peer review leads to a decision affecting the status of a manuscript submitted for publication through a publication channel (e.g., status as an official publication of the channel, assignment of a DOI etc.). Rapid checks of quality or author's credentials by editors (*access review*) are not considered as “review.” *Commentary* is an informal process allowing readers to comment on a paper but with no consequences for its formal status. A *commentary channel* is a medium (e.g., a website) allowing users to comment on papers. Publication channels, publishers, review channels and commentary channels are all classified as *communication channels*, for brevity *channels*.

### Identification of innovative models of peer review

The study began with an informal literature survey, seeded by searching Google Scholar for the term “peer review” and reviewed the content of the first three pages of results. Informal contributions to the debate (e.g., blog posts) were identified, using the same search term with Google. All papers whose abstracts indicated that they described or surveyed one or more systems of peer review, or communication channels, were downloaded and read by one of the authors. Peer review processes applying to domains other than scientific papers (grants, software, management processes, clinical interventions etc.) and actual applications of specific peer review process (e.g., examples of Open Peer Commentary) were excluded. Terminology, and names of channels were extracted and added to the list of search terms, shown in Supplemental Data Table 1. The process was iterated until the search no longer produced new terminology or new channels.

### The sample

A list was created containing all communications channels identified during the literature review. The list included one series of journals (*Biomed Central Series*) and three publishers (*Copernicus, Frontiers, Hindawi*). In each of these cases, we used Scopus to identify all journals in the series or published by the publisher, and chose the one with the highest Scimago Journal Rank (SJR) in 2013 to represent them all. Thus, the *Biomed Central Series* is represented in our sample by *BMC Genome Biology, Copernicus* by *Geoscientific Model Development, Frontiers* by *Frontiers in Synaptic Neuroscience* and *Hindawi* by *Computational Intelligence and Neuroscience*.

To ensure adequate representation for subscription journals, we added the three top-ranking journals in the Scimago rankings (Scimago Lab, 2014) for “all disciplines” (*Reviews of Modern Physics, Annual Review of Immunology, Ca- a Cancer Journal for Clinicians*), “multidisciplinary journals” (*Nature, Science*, and *PNAS*), Medicine (*Nature Genetics, Cancer Cell, Annual Review of Pathology*), the “human and social sciences” (*American Political Science Review, American Journal of Political Science, American Sociological Review*), and “arts and the humanities” (*Argument and Computation, Cognition, Journal of Memory and Language*). For the sake of completeness, we also included data from two short-term experiments in peer review (listed in Supplemental Data Sheet 1 as *Nature Experiment* and *Shakespeare Quarterly (SQ) Experiment*) that are no longer in progress. The characteristics of these experiments are described later in this paper.

The web sites of the channels included in the sample were searched for information on their respective peer review processes (usually found in “guidelines for reviewers” and/or “guidelines for authors”). Wherever possible, we also examined papers made available through the channel, gathering information about the implementation of the policies announced on the web site. The information collected in this way made it possible to characterize 82 out of 89 channels in the original list. In 7 cases, it was impossible to gather adequate information, either because the channel had ceased to operate, because the web site was not functioning, or because the required information was not available in any of the documents retrieved. These channels were excluded from the final sample.

The fact that the sample was constructed using a literature survey means that it does not provide a statistically representative picture of the whole world of academic publishing: channels likely to be discussed in the literature (e.g., channels with very large numbers of publications, channels with a high public profile, channels that have adopted innovative methods of peer review) are over-represented; conversely smaller, less innovative channels with lower profiles are under-represented. Nonetheless, the sample covers a broad range of publication methods, disciplines, and business models, and includes at least one channel for each of the innovative review processes identified in the literature survey.

The final sample consists of 50 journals, 18 preprint servers or document repositories, 3 publishers, and 17 channels belonging to other categories. 28 channels are specialized in the Natural Sciences, 17 are multidisciplinary, 12 specialize in medicine, 11 in the humanities and the arts, 6 in the social sciences, 4 in mathematics and 1 in business. 47 are freely accessible over the web, 24 are accessible by subscription only, 9 are hybrid channels including both open access and subscription material. 1 adopts a delayed open access formula, in which published papers are restricted to subscribers until 6 months after publication. In 5 cases, we were unable to ascertain the necessary information.

### Characterization of peer processes

The original intention of the study was to create a simple taxonomy of peer review systems. However, subsequent analysis showed that the channels in the sample mixed and matched different aspects of peer review identified during the literature review. Individual channels were therefore classified in terms of the seven dimensions described below.

1. When review takes place
  - No review
  - Pre-publication review,
  - Post-publication review,
  - Mixed processes (review takes place in several phases before and after publication)
2. What is assessed
  - No review
  - Non-selective review: a review process in which reviewers are instructed to limit their assessment to issues of scientific quality, without considering importance, novelty or potential impact
  - Selective review: a review in which reviewers are instructed to consider the importance, novelty and potential impact of a paper, as well as its scientific quality
3. Who are the reviewers
  - Editors
  - Reviewers selected by the editor(s)
  - Reviewers proposed by authors
  - The community (readers of the publication channel–sometimes a subset of readers)
4. Anonymity of authors
  - Double-blind: reviewers are blinded to authors' identities
  - Single-blind: authors identities are revealed to reviewers
5. Anonymity of reviewers–open review
  - Anonymous: reviewers identities are concealed throughout the review process
  - Open: reviewers identifies are revealed to authors and, in some cases, are published together with the article they have reviewed
6. Interaction
  - No: reviewers review articles independently without interacting with editors, other reviewers or authors
  - Reviewers only: reviewers discuss with other reviewers (and possibly editors) before finalizing their reports
  - Reviewers and authors: reviewers discuss with other reviewers and authors in a fully interactive process
7. Reader commentary
  - No: the channel provides no facilities for readers to comment on articles
  - In channel: the channel provides facilities for readers to comment to the articles it publishes
  - Out of channel: the channel provides facilities for readers to comment on articles published through other channels.

Each channel was classified in terms of the variables listed above. Entries were checked by both authors. The full classification data can be found in Supplemental Data Sheet 1.

### Importance of different channels and their peer review models

The quantitative importance of different channels was assessed in terms of the total number of articles published by the channel from its launch until February 2015, and the total number of articles published in 2014. Numbers were estimated by counting the number of hits from a Scopus search with the ISSN of the channel or its name. Values obtained in this way were validated against the exact figures for all Frontiers journals, which were available to the authors. In most cases, the Scopus results lay within 5% of the exact value. In cases where a journal or repository was not indexed in Scopus, values were estimated from data appearing on its web site and/or from search results in Google Scholar. Given this variety of methods, values cited later in this paper should be regarded as rough estimates. However, the difference between maximum and minimum values spans six orders of magnitude. Differences on this scale provide a useful indication of which innovations are having the greatest impact.

In addition to these quantitative measures, the study analyzed the formal and informal literature collected during the literature review to identify experimental, observational studies and specific cases offering insights into the strengths and weaknesses of particular forms of peer review. Results appearing in the primary literature, were supplemented with opinions and factual information from the blogosphere, news reports etc.

### Classical peer review in the sample

Of the 81 channels of communication in the final sample, 27, nearly all subscription journals, use “classical peer review,” as defined by the formal criteria defined earlier. Virtually all the Open Access Journals have introduced some kind of innovation. Given that the sample is deliberately biased toward innovative forms of review, it is probable that the sample underestimates the proportion of channels using classical peer review. Despite this bias, the composition of the sample clearly shows the continuing importance of classical peer review in the scientific publishing ecosystem.

## Innovative practices

### When does review take place?

#### Immediate publication with no formal review

One of the most important trends of the last 20 years has been the increasing tendency of authors to by-pass the delays, biases, unreliability and restrictions associated with classic peer review, by publishing their work directly on preprint servers, which they also use to publish materials not suitable for submission to journals (technical materials, course materials, presentations, figures, datasets) and to *republish* materials that have already appeared elsewhere (papers on subscription journals protected by pay-walls, old papers that have never been available in digital format, books).

##### Non-commercial preprint servers

The best-established channel for self-publication of scientific papers is through non-commercial preprint servers (Ginsparg, 1994, 2011; Tomaiuolo and Packer, 2000): electronic repositories, where authors can deposit papers, which are made available to users almost immediately, sometimes but not always after an “access review” to avoid “crackpots” and/or to check authors' credentials (Fitzpatrick, 2009). The most popular preprint servers are mirrored on many different sites. This guarantees that valuable data are protected against incidents affecting a single site, and makes it easier for users to efficiently upload and retrieve papers.

The oldest is *ArXiv* (http://arxiv.org/), created by Paul Ginsparg of Cornell University in 1991 and initially designed to host papers in high-energy physics (Ginsparg, 1994). *ArXiv* has been hugely successful, with the number of annual submissions that has increased from 304 in 1991 to 97,517 in 2014 (calculated from data in ArXiv, 2015a). At the time of writing (February, 2015), *ArXiv* hosts 1,013,470 preprints. It has also increased its disciplinary scope to cover most areas of physics, mathematics, and computing and even biology (though numbers of submissions in these disciplines are low).

In the early years following its creation, most of the papers hosted by *ArXiv* were preliminary versions of articles that the authors made available for community discussion and criticism before submitting them to traditional journals. Today, however, *ArXiv* has taken on a second role as a primary channel of scientific communication, which authors use to publish papers they never submit elsewhere (Fitzpatrick, 2009).

*ArXiv's* success has prompted academics to set up other preprint servers, with roughly equivalent functionality and operating procedures, though sometimes with even less “filtering.” The majority of these services cover specialized areas of mathematics and physics and contain a relatively low number of papers. Examples in the sample include the *K-theory Preprint Archives* (http://www.math.uiuc.edu/K-theory/), which has ceased to accept new preprints (964 papers), the *Linear Algebraic Groups and Related Structures Preprint Server* (http://www.math.uni-bielefeld.de/lag/) (548 papers), the *Mathematical Physics Preprint Server* (http://www.ma.utexas.edu/mp_arc/mp_arc-home.html) (5701 papers), *Preprints on Conservation Laws* (http://www.math.ntnu.no/conservation/) (834 papers) and the *Real Algebraic and Analytical Geometry Preprint Server* (http://www.maths.manchester.ac.uk/raag/) (352 papers). More extensive lists can be found at (Auburn University, 2014; University of Wollongong, 2014; Wikipedia, 2014; Mathematics on the Web, 2015). The literature suggests that uptake of these services, like that of *ArXiv* itself, was favored by a strong tradition in mathematics and physics of distributing preprints, in paper format, prior to publication (Ginsparg, 1994). Other factors favoring uptake may have included high levels of IT know-how, and lack of opportunities for commercial exploitation.

Probably the second largest preprint server after *ArXiv* is the official Chinese server *Scientificpaper online* (http://www.paper.edu.cn/en), which covers the full range of academic disciplines, with an unusual emphasis on “electric, communication, and autocontrol technology.” At the time of writing, the server contained just 6748 papers in English (Sciencepaper Online, 2015). However, Scopus indexes approximately 86,000 papers on the server, mostly in Chinese.

Although preprint servers have had the most impact in mathematics and physics, they have also spread to other disciplines, including social science, cognitive science, and biology (Kaiser, 2013), but not, as far as the authors can tell, medicine. Examples in the sample include the *Social Science Research Network* (http://www.ssrn.com/en/), *Cogprints* (http://cogprints.org/), which specializes in psychology, cognitive science and computing and *BiorXiv*, designed for biologists. These services receive far fewer submissions and are indexed more rarely than equivalent services in mathematics and physics. At the time of writing, data for SSRN was not available; *Cogprints* contained 4167 papers (Cogprints, 2015), and *BiorXiv* just 1616 (bioRxiv, 2015). Many of these papers had already been published elsewhere, and may have been deposited to comply with funding agency open access policies. These data suggest that neither of these services has become a genuinely important channel for scientific publication. This conclusion is not changed by the fact that a significant number of biologists publish on *ArXiv* (9653 articles in the period 1991–2014) (ArXiv, 2015b) with interest reported to be particularly high in evolutionary and population genetics (Haldane's sieve, 2015).

To summarize, pre-publication servers have become an important channel for scientific publication. Although most of the preprint servers in the sample have seen little recent growth in submissions, submissions to *ArXiv* more than compensate for the weakness of smaller channels (see Figure 2).

The main impact has been in physics, mathematics, and astronomy. In other disciplines, particularly biology and medicine, pre-print servers have had only a very limited impact. This suggests that in most disciplines preprint servers pose only a limited threat to classical peer review. However, publishers will find it difficult to promote alternative publication models in disciplines where *ArXiv* is strong.

##### Preprint services from journal publishers

In recent years, a few journals have also begun to offer preprint services. The two examples in the survey sample are *Nature Precedings* (http://precedings.nature.com/), which ceased to accept new manuscripts in 2012 (Nature Precedings, 2015), and *PeerJ Preprints* (https://peerj.com/about/publications/%23PeerJ-PrePrints), which is still in operation. Both services focus on the biomedical sciences.

Before it ceased operations, *Nature Precedings* published 5058 papers (calculated using advanced search function in Nature Precedings, 2015). This is significantly more than *BiorXiv*, the main non-commercial preprint server for biologists, and roughly equivalent to the number of biological papers published by *ArXiv* over a much longer period. At the time of writing, the *PeerJ Preprints* archive contains 1015 papers (Cf. https://peerj.com/).

These numbers suggest the sites were at least partially a success. However, they do not seem to provide an attractive model for publishers. When *Nature Precedings* ceased operations, the Nature Publishing Group stated that “technological advances and the needs of the research community have evolved to the extent that the *Nature Precedings* site is unsustainable as it was originally conceived” (Nature Publishing Group, 2015). This experience, and the lack of other offerings in this area are evidence that traditional publishers find it difficult to compete with non-commercial or non-traditional commercial actors in this segment of the market.

#### Immediate publication with post-publication review

One way of eliminating the delays but conserving the advantages associated with classical peer review is to publish submitted papers immediately (usually after a rapid “access review”) and to perform formal peer review *after publication*. If names of reviewers and reviewer reports were published, as in so-called Open Review systems (see below), such a system could give rise to a completely new system of Open Evaluation, as proposed in Kriegeskorte (2012). To date, no journal has fully implemented these proposals. Existing systems of post-publication review are summarized below.

**In-channel post-publication review** can be defined as a review process that is managed by the same channel that hosts the article under review. It is relatively rare (just 3 examples in the sample). The most important examples (all in the sample) are discussed in-depth in (Pöschl, 2012).

To the knowledge of the authors, the first journal to adopt in-channel post-publication review was *Electronic Transactions in Artificial Intelligence (ETAI)* (http://www.etaij.org/), an online journal, launched by Erik Sandewall, in 1997 (Sandewall, 1997; Fitzpatrick, 2009; Pöschl, 2012). When ETAI received a submission from an author it was submitted to a brief access review. If it passed the review, it was published immediately, in pre-print format. Publication was followed by an open interactive discussion in which any reader of ETAI could participate (there were no designated referees). In a second phase of the process, articles were examined by anonymous referees, who made the final recommendations used by the editors in their publication decision. The experiment does not appear to have been successful. ETAI ceased publication in 2002, having published just 100 articles. The website for the journal was last updated in 2006.

A better-known, and more successful example of in-channel post-publication review is *Atmospheric Chemistry and Physics (ACP)* (http://www.atmospheric-chemistry-and-physics.net/), a journal published by *Copernicus Publications* (http://publications.copernicus.org/). In 2001, *ACP* introduced a 2-stage peer review process, which was later imitated by sister journals, also published by the European Geosciences Union, and by *Copernicus*. Following rapid pre-screening (access review), to check that they meet normal standards for academic publishing, papers submitted to *ACP* appear immediately on the journal's website, where they are given the status of a “discussion papers.” This establishes the authors' precedence and makes their findings immediately available to the scientific community. Once the paper has been published on the web site, the paper is assigned to peer reviewers, following the same procedures normally used for pre-publication review. There ensues a public discussion between the reviewers and the authors, in which other interested members of the scientific can also participate. This discussion, which lasts 8 weeks, constitutes the first phase of the review process. It is followed by a second phase of non-public revisions and review following the model used in traditional journals. If this leads to acceptance of the paper, it is published on the main journal (Koop and Pöschl, 2006). Comments made during the review process, and the authors' replies are published alongside the main article.

Rejection rates are low (about 20%). In this way, *ACP* avoids the major delays associated with submission to multiple journals in sequence. The editors report that the journal's two-phase review process has contributed significantly to quality assurance. Claimed advantages include the creation of disincentives for the substandard manuscripts (authors do not want to have poor quality work identified as such in public) and the possibility offered reviewers to claim authorship for their contributions (Koop and Pöschl, 2006). At the time of writing, Scimago ranks *Atmospheric Chemistry and Physics* fifth out of 100 journals in Atmospheric Science. Since its creation the journal, has published 6239 articles (of which 747 in 2014).

Other channels offering immediate publication and post-publication review include *F1000Research, Philica*, and the *Semantic Web Journal*, online journals that use a variety of business models. *F1000Research* publishes original research papers in biology and medicine, which are subsequently submitted to post-publication review; authors are charged a publication fee. *Philica* offers completely free immediate publication of any scientific article, again followed by a post-publication review. Following a brief access review, articles submitted to the *Semantic Web Journal* (http://www.semantic-web-journal.net/), are immediately posted to the journal's web site, and announced in a blog. The editors then solicit reviews from three reviewers, and invite users of the site to submit their own reviews—all of which are published on the web site. After 8 weeks, the editors decide whether or not the paper should be officially published on the journal, based on the reviews they have received (Semantic Web Journal, 2015). None of these channels appears to have a major impact. At the time of writing, *F1000Research* had published a total of 713 papers (of which 351 in 2014), *Philica* 422 (data for 2014 not available) and *Semantic Web Journal* 183 (42 in 2014). The titles appearing on the *Philica* web page suggest, furthermore, that not all documents published through this channel meet normal criteria for scientific publication.

**Out of channel post-publication review** is a service offered by a channel that provides quality assured reviews of articles originally published on other channels (e.g., preprint servers)—an idea originally proposed by Juan Miguel Campanario (Campanario, 1997), and subsequently taken up by other authors (Smith, 2000; Moyle and Polydoratou, 2007; Brown, 2010), who often refer to these channels as “overlay journals.” One of their key characteristics is that they aim to provide a high quality service, in some way equivalent to traditional pre-publication review. In general, therefore, they use formal guidelines for evaluation criteria, the selection of reviewers, the duration of the review process etc. This formality distinguishes them from services such as ResearchGate's *Open Review service* (https://www.researchgate.net/publicliterature.OpenReviewInfo.html) that allow any individual user to write a review, and services that allow readers to comment on a paper after publication or give it a rating, without enforcing any kind of formal procedure.

*F1000 Prime* (http://f1000.com/prime), founded in 2009, publishes reviews of individual papers in the biomedical sciences. The reviewers are members of the *Faculty of 1000*, who recommend the paper to the service's users, and who sign their reviews. The reviews are not indexed by major indexing services. However, the search function on the *F1000* web site finds 124,883 reviews (of which 6596 in 2014). This suggests that the service is attracting significant numbers of reviewers and readers, and that it is beginning to take on a significant role.

*F1000 Prime* is complemented, by *F1000 Prime Reports*—a series of review reports focusing on emerging themes in biology and medicine, which F1000 launched in 2013. At the time of writing, the service has published 682 reports of which 121 in 2014.

Taken together, these results are evidence for the viability of *F1000's* business model. The model appears to offer advantages to authors, who benefit from a transparent review process, and the publicity it provides for their work, readers, for whom F1000 reviewers can facilitate the selection of articles to read, and reviewers who receive (limited) public recognition for their work.

Another channel with a different business model is *Science Open Research*. Science Open Research charges authors a fee to take papers that have already appeared on public preprint servers, publish them in a new context, provide a post-publication review, and provide support for reader comments. Although the journal has attracted skeptical commentary (Scholarly Open Access, 2014), it is indexed more than 3000 times by Google Scholar. This suggests there is demand for this kind of service. Nonetheless, it is clear that *Science Open Research* and the other smaller players in the sample have a very small impact on the overall market.

#### Multiphase processes

Two channels in the sample use multiphase review processes that mix features of pre-publication and post-publication review.

The first is *Frontiers in Synaptic Neuroscience*, a “tier 1” journal in Frontiers' tiered publication system (http://www.frontiersin.org/about/tieringsystem). In this system, original research papers addressing a specialist audience (*tier 1 articles*) undergo normal pre-publication review. Then, in the 3 months following publication, Frontiers records the number of times each paper is viewed and downloaded. Finally, following a quality check by a scientific editor, the authors of the 10% of papers with the most views and downloads, are invited to transform their paper into a “Focused Review” for a more general academic audience. Focused reviews are published in Frontiers “field journals” (tier 2 journals). Like original research articles, Focused Reviews undergo pre-publication review. At the time of writing, *Frontiers* has published 30,000 articles, of which 256 “climbed a tier” to become Focused Reviews.

The second channel in our sample to use a multi-phase process is the *Journal of Interactive Media Education (JIME)*, (http://www-jime.open.ac.uk/) which began publication in 1996, with a review model that mixed pre-publication and post-publication review. In the original *JIME* process, submitted papers were first submitted to a “private open peer review”—in which authors and reviewers interacted freely using a private website. If a paper was judged to be of sufficient quality it was then published as a preprint, which became available for public community review by interested members of the relevant community. The discussion during the public review was published together with the final version of the paper [information retrieved from an archived version of the *JIME* web page for 1996–7 (Journal of Interactive Media in Education, 1996)]. The authors have not been able to retrieve any useful information concerning the success of this system, which is no longer mentioned on the journal's web site (Journal of Interactive Media in Education, 2014). The journal has hosted only 140 papers in the 17 years of its existence. The survey found no comparable systems. It seems, therefore, that the *JIME* model, while interesting, has had no lasting impact.

#### Portable review

In many cases, a journal editor may reject a paper after it has been reviewed, but suggest that it might be suitable for publication in another journal. Normally this means that the paper goes through a second round of review, delaying publication.

One way of reducing the delay is by instituting a process of *portable review* in which reports from the review by the first journal are passed on to the editor of the second. In 2008, a number of neuroscience journals agreed to accept manuscript reviews from one another. Although the experiment met with limited success (Van Noorden, 2013), several journals have begun to share reviews with other journals managed by the same publisher. Publishers involved in such schemes include *Biomedcentral* (http://www.biomedcentral.com), the *British Medical Journal* (http://www.bmj.com/), *Elife* (http://elifesciences.org/), and the *Nature Publishing Group* (http://www.nature.com/).

To the knowledge of the authors, there has never been a formal study of how many papers have benefited from portable review, and how much it has reduced delay in publication. In one specific case, the managing director of BMC reports that *BMC Genome Biology* accepts only 10% of submitted papers but passes on 40% of the rejected papers to other BMC journals (Van Noorden, 2013).

An alternative approach, proposed by *Peerage of Science* (http://www.peerageofscience.org/) and *Rubriq* (http://www.rubriq.com/) is for authors to submit their papers for review by a commercial review service, *before* submitting them to a journal or before allowing the service to submit the paper on the author's behalf. *Peerage of Science* and *Rubriq* have different business models. Peerage of Science receives revenue only from journals and publishers and is free for authors and reviewers, collaborating with a significant number of journals and publishers who welcome links to Peerage of Science reviews in manuscripts submitted to them, and which may use the reviews their editorial decision-making. Examples in the sample include *PeerJ, PLOS Biology*, and *PLOS ONE*. *Rubriq*, by contrast, charges authors a fee, and appears to have fewer collaborations with journals. Both services use a formalized review process, including some kind of “standardized score card.” However, their web sites provide no data about how many authors are using their services. It is safe to conclude that pre-submission review has yet to make a major impact.

### What is assessed?

Paper-based journals, with high marginal costs, low page budgets and a subscription-based business model are necessary selective in choosing the papers they publish. The rise of online and Open Access Publishing relaxed some of these constraints. In particular, online publishing and the introduction of author fees made it economically feasible (and advantageous) for online, open access journals to remove restrictions on the number of articles they published and to be less selective in selecting the articles.

Some of the first Open Access journals adopted highly selective review policies, as testified by their publication record. For example, *BMC Medicine* (*Biomed Central's* flagship journal) has published only 1127 papers since its foundation in 2003, which 175 in 2014. However, many publishers were less selective. *The BMC Series* (BMC) for example, has published 94,000 papers.

In the early years of Open Access publishing, these shifts in reviewing practice were not reflected in reviewer guidelines. Even today, in fact, reviewer guidelines for journals in the *BMC Series* and for journals published by *Hindawi* are very similar to those issued by high impact subscription journals. However, other journals and publishers made the change explicit.

In 2006, *PLOS ONE* (http://www.plosone.org/) and in 2007, our own organization, *Frontiers* (http://www.frontiersin.org/), independently introduced what some authors now call “non-selective” or “impact-neutral review” (Ccanz, 2013). *Frontiers* made a new conceptual distinction between *review*—a formal process aimed to guarantee that articles met strong standards for scientific quality—and informal *evaluation*, in which the importance or otherwise of particular results, interpretations and hypotheses emerges from the gradual construction of consensus within a scientific community. To translate this concept into practice, it adopted a system of “objective review” in which reviewer reports are based on a standardized questionnaire and the only acceptable ground for rejecting a paper is objective error (Frontiers in Neuroscience, 2015). In other words, papers are no longer rejected because they are not sufficiently important or novel or because they challenge mainstream opinion. *PLOS ONE* adopts similar criteria. According to the *PLOS ONE* reviewer guidelines, the journal uses peer review, exclusively to determine “whether a paper is technically sound and worthy of inclusion in the published scientific record” (PLOS ONE, 2015a). Critical to both channels' approaches is the absence of any attempt to limit the number of papers accepted for publication.

**Figure 3:**
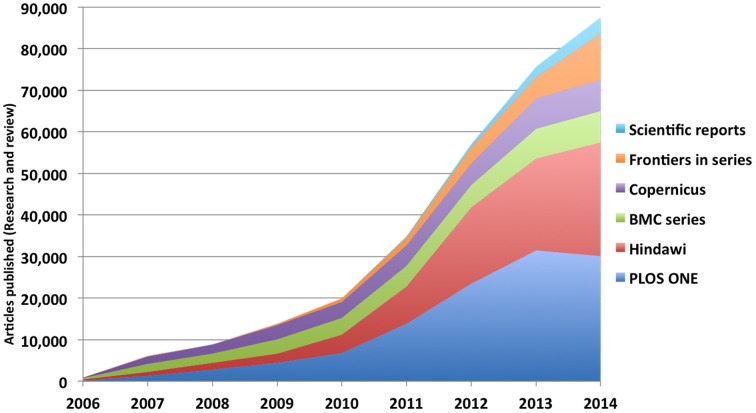
**Cumulative number of review and research articles published in series or by publishers explicitly or implicitly adopting non-selective review**. Scientific Reports: data calculated from year by year lists of papers at (Scientific Reports, 2015); Frontiers**—**internal data; BMC: results from Scopus search for “articles,” “review,” and “articles in press” (February 18, 2015); Hindawi—results from search for “research articles” and “review articles” at http://www.hindawi.com/search/ (February 18, 2015). PLOS ONR**—**results from search for “research article” and “systematic review” at http://www.plosone.org/search/advanced?noSearchFlag=true&query=&filterJournals=PLoSONE (February 18, 2015). Data for BMC series and Hindawi includes some journals that use selective review. The numbers of papers published by these journals is too small to significantly affect the results.

In the last 4 years, many other Open Access journals have adopted similar systems or have moved in the same direction. Examples in the sample BMC's *Biology Direct* (http://www.biologydirect.com/), *F1000 Research* (http://f1000research.com/), *GigaScience* (http://www.gigasciencejournal.com/), the Journal of Negative Results in Biomedicine (http://www.jnrbm.com/), *OpenBMJ* (http://bmjopen.bmj.com/), *PeerJ* (https://peerj.com/) and *ScienceOpen Research* (https://www.scienceopen.com). The *Hindawi* group (http://www.hindawi.com/), while not explicitly embracing non-selective review, also expects its acceptance rates to increase (Loy, 2011). Considering these trends together, it is clear that non-selective review is having a major impact on the publishing scene. Using data from different sources, we estimate that, in 2014, major publishers and journal series adopting non-selective review published more than 90,000 papers (see Figure 3). Interestingly, all the journals in the sample that have adopted non-selective review focus exclusively or primarily on biomedical science—an area which places great emphasis on high quality experimental results, and in which rapid access to reliable data is of critical importance for practitioners. To the knowledge of the authors, journals in other disciplines have not yet adopted the system. However, new *Frontiers* journals in Engineering, Social Sciences and Humanities will adopt the same non-selective criteria used in other *Frontiers* journals.

As might be expected, journals that have adopted the new selection criteria have high acceptance rates. *Frontiers* has accepted roughly 80% of papers submitted to it since it began operations (unpublished data). *PLOS ONE* states on its web site that its acceptance rate is 69% (PLOS ONE, 2015b). Proponents of the new procedures have suggested they could have numerous advantages. Lower rejection rates mean that more manuscripts are published and that fewer authors have to resubmit their manuscripts to multiple journals before they accepted—in other words it speeds up the publication process. “Non-selective review” should make it less likely that journals reject valuable work because it is out of line with mainstream opinion, or because it is unlikely to attract a broad readership. Presumably, it also provides fewer opportunities for bias (Ccanz, 2013).

To date, however, there has been no objective study of the way non-selective review has changed patterns of scientific publication. It is not known, for instance, how far it has been successful in speeding up the publication process, or in making publishing more accessible to female authors, authors from developing countries, and authors or from relatively unknown institutions. Nor is it known how far it succeeded in promoting the publication of controversial and negative results and replication studies.

There have also been no published studies of the effect of high acceptance rates on scientific quality. If classical review genuinely succeeds in selecting the best papers, one would expect the quality of papers in journals using non-selective review to be lower. This is a topic for future research. What is certain is that the impact of non-selective review is potentially very large. In purely numerical terms, *PLOS ONE* has published over 131,000 papers since its foundation in 2006 (of which 33,102 in 2014), Since opening for submissions in 2007, *Frontiers* has published over 30,000 (of which 11,130 in 2014 alone).

### Who are the reviewers and who chooses them?

#### Review by editors (no external reviewers)

In two of the channels in the sample—the *Harvard Business Review* (http://hbr.org/) and *Kairos* (http://kairos.technorhetoric.net/), an open access journal in the humanities that explores “the intersections of rhetoric, technology, and pedagogy” (Kairos, 2015)—review is performed directly by the editors of the journal. In the case of *Kairos*, editors are reported to engage intense online discussions before reaching their decisions, often writing thousands of words about submissions (Mole, 2012). This review process is highly unusual, and is obviously unsuitable for channels handling very large numbers of submissions. Nonetheless the high reputations of the *Harvard Business Review* and *Kairos* suggest that, in some cases, it can be extremely successful.

#### Author-selected reviewers

Many subscription and Open Access journals allow authors to suggest reviewers for their papers, and/or to list reviewers they would not consider as suitable. In most cases, journals will attempt to take account of authors' wishes—particularly with respect to names they prefer to exclude from the review process. In general, however, the final choice is at the discretion of the editor(s).

A number of journals allow authors to play a greater role in reviewer selection—avoiding some of the problems that arise when reviewers are chosen by editors. Probably the most important is *Proceedings of the National Academy of Sciences* (PNAS, 2015) (http://www.pnas.org/). Although *PNAS* maintains editorial discretion over the final choice of reviewers, it requires authors to nominate “three appropriate Editorial Board Members, three members of the National Academy of Sciences (NAS) who are expert in the paper's scientific area and five qualified reviewers.” In cases where a paper “falls into an area without broad representation in the NAS, or for research that may be considered counter to a prevailing view or far too ahead of its time to receive a fair hearing,” authors may also ask a member of the NAS to oversee the review process (Proceedings of the National Academiy of Sciences of the United States of America). *PNAS* is particularly important because of the large number of papers it publishes (113,592 papers of which 33,102 in 2014).

A similar system has been adopted by Biomed Central's *Biology Direct* (http://www.biologydirect.com/), which, unlike PNAS, adopts a non-selective review process. Authors for the journal select their own reviewers from among the members of the editorial board, and the journal publishes any paper that at least three members of the board agree to review. Reviewers' comments are signed and published alongside the paper itself. The journal adopts an extremely liberal acceptance policy: papers with critical or negative reviews are still published, though authors are free to withdraw them, ahead of publication (Koonin, 2006).

#### Open peer commentary

One of the oldest, best-established ways of avoiding the problems associated with small numbers of editor-chosen reviewers is so-called “Open Peer Commentary”, a review procedure in which original papers (“target papers”) are published side by side with in-depth written commentary (often 1000 or 1500 words), usually from invited experts in the relevant field, but sometimes from other members of the scientific community. To the knowledge of the authors, the first journal to adopt the procedure was *Current Anthropology* (http://www.press.uchicago.edu/ucp/journals/journal/ca.html), which has been using it since the journal's foundation by anthropologist Sol Tax in 1959. In the original version of the system, allegedly modeled on the participatory decision-making practices of certain native American nations, editors assigned manuscripts to a large number of referees (sometimes more than a hundred) whose comments were communicated to the author(s), who revised their articles accordingly. Reviewers' comments and authors' replies and acknowledgements were published alongside the revised version of the article.

Over time, this system gradually evolved into a more efficient, multiphase system. The revised system, which is still in use (The University of Chicago Press Journals, 2014), involves large numbers of referees. However, it introduces a clear separation between the initial phase of the process, in which the exchange between authors and referees is informal, and a second phase in which reviewers write formal commentaries and authors write formal replies, which are published with the final version of the paper (Harnad, 1979). The system appears to be successful. Fifty-five years after its foundation *Current Anthropology* has a strong reputation, despite the fact that it publishes relatively few articles (1376 articles of which 108 in 2014). It is plausible that the low number of articles is linked to the complexity and cost of the journal's review process.

Stevan Harnad, an eloquent advocate for the reform of classical peer review, who had already written about *Current Anthropology* (Harnad, 1979), went on to found *Behavioral and Brain Sciences* (BBS) (http://journals.cambridge.org/action/displayJournal?jid=BBS), a traditional paper journal with 5530 publications, of which 264 in 2014. Harnad also founded *Psycholquy*, an online journal, no longer in operation. Both of these journals adopted a form of Open Peer Commentary modeled on *Current Anthropology*. An unusual feature of *Psycholquy*, which was dedicated to psychology and related fields, was the attempt to make the review process extremely fast—enabling authors to engage in an on going conversation with reviewers while their ideas were still fresh (Harnad, 1990, 1991). This idea has been taken up by other more recent journals that have implemented forms of “interactive review” or “developmental editing.” Interactive review and developmental reviewing will be discussed in greater depth later in this paper.

The only other journals in the sample that have adopted Open Peer Commentary are the *American Journal of Bioethics* (http://www.tandfonline.com/loi/uajb20#.U7JvS5SSxBM) and the *Journal of Media Education* (JIME), which later abandoned the system, possibly because it only attracted a small number of publications (Pöschl, 2012).

The low number of publications in *Current Anthropology*, the failure of *Psycholquy*, and *JIME's* decision to abandon open peer commentary, show the difficulties inherent in a review process that requires major effort from authors and large numbers of reviewers. This means that Open Peer Commentary is unlikely to spread widely. Nonetheless, the experience of *Current Anthropology* and *BBS* show that, when well implemented, it can be an extremely valuable tool.

#### Community/public review

One strategy to resolve some of the problems associated with classical peer review is to institute a review process open to all members of the scientific community. In principle, such a process could eliminate problems arising from bias in editors' selection of reviewers, and the unreliability of review processes that use only a small number of reviewers.

None of the journals in the sample uses such a process on its own. However, two—*ETAI* and the *Semantic Web Journal*—combine community review with classical pre-publication review and two more—*Nature* and the *SQ* (http://mcpress.media-commons.org/ShakespeareQuarterly_NewMedia/) have conducted experiments to test the concept.

The experience of *ETAI* (no longer in operation) and the *Semantic Web Journal* has been described earlier in this paper. In both cases, the journals combined classical review by editor-chosen reviewers with community review by users of the journal website. In the case of *ETAI*, the classical review process *followed* the community review. In the case of the *Semantic Web Journal*, the two processes were carried out in parallel.

*Nature's* experiment lasted from June to December 2006, again combining review by editor-chosen reviewers with a community process. During the experiment, authors submitting manuscripts were given the option of making them available for community review. In this case, *Nature* published the manuscript on its website inviting public commentary from users, which the editors were supposed to take into consideration, alongside reports from anonymous reviewers, when they made their publication decision. In December 2006, *Nature* ended the experiment, judging that it had not been a success. Only 5% of authors had opted into the community review process, and only 54% of manuscripts had attracted substantial commentary. Furthermore, most of the comments received were not sufficiently substantial to be useful to editors (Nature, 2006a; Fitzpatrick, 2009). Critics suggested that *Nature's* decision to make author participation that making the experiment optional had weakened its validity and this may be correct (Brownlee, 2006; Campbell, 2006). However, the problems in attracting reviews from readers were genuine. These may have been due to readers' reluctance to publicly criticize colleagues or simply to lack of interest. In either case, they point to the real obstacles facing journals wishing to use community review, at least in the life sciences.

The *SQ* experiment, the first test of community review in the humanities, seems to have been more successful. The experiment, which lasted from March to May 2010, again allowed authors to opt into the process and again combined review by invited reviewers with commentary by interested readers. However, user participation seems to have been much higher than in the Nature experiment. Kathleen Rowe, who was guest editor of *SQ* at the time, reports that

“Forty-one participants (including the submitters, Editor, and guest editor) posted more than 350 comments, making for a lively exchange. The journal's open review pages on MediaCommons were accessed over 9500 times. Commenters self-identified; a majority were those at tenured rank. Their comments addressed stylistic, historical and theoretical matters, and ranged from passing responses to sustained engagement and challenge. Authorial revisions in response to these comments were meticulous and in several instances very substantial. (…) Along with a core of well-known Shakespeare and media scholars, critics from related fields took note and weighed in thoughtfully, including digital humanities experts. These voices provided an interdisciplinary range and perspective not available in a traditional *SQ* review. –” (Rowe, 2010)

This report suggests that, in the right conditions, community review can indeed be valuable. However, *SQ* has not repeated its experiment. For the moment, community review, while an interesting concept, has yet to acquire a real role in the scientific publishing ecosphere.

#### Other systems

One high impact journal in our sample—*Anti-Oxidants and Redox Signaling* (*ARS*) (http://www.liebertpub.com/overview/antioxidants-and-redox-signaling/4/)—has introduced an unusual review procedure, which the publisher calls “rebound review.” The *ARS* review process begins with a phase of conventional peer review by editor-chosen reviewers. If the results of the review are negative, authors may then request a second review by recognized external experts they propose themselves. If at least four recommend acceptance of the paper, the editor will consider their reports in deciding for or against publication (Sen, 2012). In principle, this procedure could help authors to correct mistaken editorial decisions caused by poor choice of reviewers, reviewer bias or the use of a low number of reviewers. However, critics have suggested that a conventional appeals process, as practiced by many journals, could have achieved the same result, faster, and at lower cost (Ryter and Choi, 2013). *ARS* is the only journal in the sample to provide this kind of redress procedure. To date, its impact appears to be limited.

### Author anonymity (partially blinded vs. double blinded review)

Different disciplines have long followed different practices concerning author anonymity during the review process. In the humanities, the arts, and the social sciences, authors are usually anonymous. In the natural sciences, by contrast, there is normally no attempt to conceal authors' identity. Of the 56 channels in the sample that use some form of review, 40 reveal authors' names to reviewers (single blind review); 11 channels, mainly in the arts, the humanities and the social sciences, do not (double blind review). In 2 cases, authors can opt between single and double blind review. As expected, the majority of channels that use single-blind review are in medicine and the natural sciences. Significantly, they include series, publishers, and journals with very large numbers of publications (*the BMC series, Frontiers, PLOS ONE*).

At the time of writing, the use of double-blind review is in flux. On the one hand, the *American Economics Association* has announced that it is abandoning double blind review, partly for reasons of cost, partly because search engines have made it too easy for reviewers to identify authors (Jaschik, 2011). On the other, double-blind review is becoming more common in the hard sciences. The sample includes two journals in the natural sciences and computing—*Behavioral Ecology* (http://beheco.oxfordjournals.org/) and *IEEE Communications Letters* (http://www.comsoc.org/cl)—that use double blind review. Two additional journals—*Nature Climate Change* (http://www.nature.com/nclimate/index.html), and *Nature Genetics* (http://www.nature.com/ng/index.html) are running experiments in which authors can opt for double blind review, and an additional journal (*Conservation Biology*) is reported to be considering the system (Darling, 2014; Nature Climate Change, 2014).

Proponents of double blind review produce evidence that single blind review leads to bias against female authors and authors from low status position institutions or countries, and suggest that double blind review can reduce these biases (Snodgrass, 2006; Clarke, 2008; Cressey, 2014). Opponents suggest that knowing authors' names allows reviewers to ask appropriate questions (e.g., differentiating between poor writing and bad experimental technique), to compare a paper against previous work by the same author, and to identify possible conflicts of interest (Clarke, 2008). They also argue that double blinding provides only an illusion of anonymity, and that reviewers can offer guess authors' identities from the content and style of their papers or from the references they use. These suggestions are supported by studies showing that in many cases authors can be identified using citation data alone (Hill and Provost, 2003) and that masking of authors' identities is effective at best in 75% of cases (Snodgrass, 2006).

Findings from studies of journals that have actually adopted the practice are non-conclusive. For example, Budden and colleagues show that the introduction of double-blind review by *Behavioral Ecology* was followed by an increase in papers with female first authors (Budden et al., 2008). However, a second paper reports that female first authorship also increased in comparable journals that do not use double blind review (Webb et al., 2008). A study by Madden and colleagues shows that the adoption of double-blind reviewing in the SIGMOD conference led to no measureable change in the proportion of accepted papers coming from “prolific” and less well-established authors (Madden and DeWitt, 2006), but a later study contests this conclusion (Tung, 2006).

### Reviewer anonymity vs. open review

In classical peer review, reviewers are anonymous. The alternative is some form of “open review” in which reviewer names are revealed to authors (sometimes only authors whose papers are accepted) and (usually) published. 26/57 channels in the sample adopt review processes meeting these criteria. In at least one case (*Frontiers in Synaptic Neuroscience*), the reviewers of rejected papers maintain their anonymity (Frontiers in Neuroscience, 2015)—a rule that defuses the common argument that open review could prevent reviewers (especially junior reviewers) from openly expressing criticism of (more senior) colleagues. Another channel (the *BMJ*) adopts the opposite rule (authors are informed of reviewers identities but the reviewers names are *not* published). In many cases, channels that publish reviewers' names also publish their full reports and their interactions with authors. This practice is followed by many important channels that use pre-publication review by editor-chosen reviewers (e.g., the *BMC series, PeerJ*). Yet another set of channels (*American Journal of Bioethics, eLife, EMBO Journal*, and *Philica*) practice a different form of Open Review, publishing reviewer reports but maintaining the anonymity of reviewers.

Considering its deliberate bias toward journals with innovative forms of peer review, it is probable that “open review” is over-represented in the sample. It is clear, however, that it is a significant presence. The impact is less clear. Experiments comparing the performance of anonymous reviewers to that of reviewers who sign their reviews have consistently failed to show that the quality of reviews from reviewers who sign their reviews is significantly better (or worse) than that of anonymous reviews (McNutt et al., 1990; Van Rooyen et al., 1999b, 2010). However, each of these studies involved reviewers from a single medical journal. Furthermore, the validated Review Quality Instrument (Van Rooyen et al., 1999a) used in two of the studies, was not designed to address issues of bias and ethics. To the knowledge of the authors, there are no observational studies to date that compare the performance of anonymous and open review on these critical issues.

### Interaction

Criticism of classical peer review processes have led two channels in the sample (*EMBO Journal and eLife*) to make interaction among pre-publication reviewers a normal part of their review process (Hames, 2014). *EMBO Journal*, for example, implements a system of cross-peer review, in which “referees are invited to comment on each other's reports, before the editor makes a decision, ensuring a balanced review process” (EMBOJ, 2015). In *eLife*, “the reviewing editor initiates an online consultation session in which each referee can see who the other referees are and what they wrote about the manuscript” (Schekman et al., 2013).

Other channels have gone further, encouraging reviewers and editors to interact with authors. In 2008, for example, *Frontiers* introduced a Collaborative Review Forum, which makes direct interactions and exchange between reviewers, authors and editors a standard procedure and facilitates constructive feedback and dialog (http://www.frontiersin.org/about/reviewsystem). Other examples in the sample include one technical journal (the *Semantic Web Journal*), one journal in psychology and related disciplines (*Psycholoquy*), and three channels addressing the humanities (*JIME, Kairos*, and the *SQ experiment*). Two (*Kairos*, and *Frontiers in Synaptic Neuroscience*) restrict access to the review to authors, reviewers, and editors. The others use public web sites. In all cases, the aim is that reviewers should help authors to improve the quality of their papers through “developmental editing.” In some cases, the interactions are intense. The editor of *Kairos* reports, for instance, that “thousands of words are written about submissions, and lengthy discussions take place – all to figure out the best content for the journal” (Jaschik, 2012)

Reports from participants are generally but not universally positive. The editor of the *SQ Experiment* reports that, “for some Shakespeareans, writing reviews with ‘someone looking over my shoulder’ was nerve-wracking and inhibiting, making total frankness impossible. Others, as we discovered, refuse to review anonymously in principle; these saw no challenges to frank assessment. In our view the majority, somewhere between these poles, achieved a successful balance.” (Rowe, 2010). To date, there have been no observational or experimental studies of interactions among reviewers or between reviewers and authors. Their actual impact on quality of review, and quality of papers remains an open question.

### Reader commentary

The long-term impact of a scientific publication depends on the way it is “evaluated” by the scientific community to which it is addressed, and it is well known that community evaluation often gives very different results from formal peer review. Historically, community evaluation was an informal process that left little trace. Today, however, technology allows part of the process to take part on the web. Informal reader commentary should nonetheless be clearly distinguished from community peer-review (e.g., the process used by *Atmospheric Chemistry and Physics*), a formal process that determines whether the article under review will be published.

#### In channel reader evaluation

Many modern channels provide facilities for readers to comment on articles that have already been through formal peer review. Among the channels in the sample that use formal review, and for which the required information was available, 24/53 provide facilities allowing users to comment on published articles. Nearly all are multidisciplinary, or specialized in the natural sciences or computer science. Similar facilities are provided by one channel in the humanities and the arts, one channel in business and none in the social sciences. The 29 remaining channels are spread across the full range of disciplines in the sample.

Of course, the mere presence of technological facilities for user commentary does not guarantee that users will use them or that their commentary will have an impact. Of articles published in *PLOS ONE* up to 2010, only about 17% attracted reader comments (Veitch, 2010). The editor of the *BMJ* has observed that while a short sports blog may attract two thousand comments, the majority of scientific articles attract no comments at all. He adds that “while there are no incentives for scientists to comment on other scientists' papers, fear of upsetting seniors, giving away good ideas, or being wrong” may act as disincentives (Smith, 2011).

#### “Out of channel” reader commentary

Scientists' apparent reluctance to use in-channel reader commentary has not prevented the emergence of channels offering facilities for informal commentary as their main service, or as part of their service. Examples in the sample include *F1000 Prime, PubMed Commons, Pubpeer.com, Mendeley*, and *ResearchGate*. *Mendeley* and *ResearchGate* present themselves as social networks for academics and repositories for documents originally published elsewhere. *Academia.edu*—another “academic social network” is also reported to be working on a “peer review and discussion” platform (Price, 2013). Much reader commentary is also published though channels not included in the sample, in particular blogs (Hames, 2014), and Twitter. Our survey suggests that at least in some cases, this discussion has made important contribution to the scientific process, usually by criticizing extraordinary claims in the formal and informal literature. One recent case is the extremely rapid debunking of the STAP technique for the manufacture of pluripotent cells, published just in January 2014 (Obokata et al., 2014). In this case, user comments on *Pubpeer.com* (Multiple Authors, 2014a; PubPeer, 2015), *ResearchGate* (Lee, 2014) and blogs (Knoepfler, 2014; Multiple Authors, 2014b) all played an important role, with comments on pubpeer alone attracting more than 25,000 views. Others include a purported proof that *P* ≠ NP (Neylon, 2010; Lipton, 2011), and subsequently retracted claims about cells that use arsenic in place of phosphorus (Redfield, 2010).

One of the channels where these comments were published (*pubpeer.com*) provides users with anonymity. However, other channels and most blogs publish authors' identities. Some commentators have argued that this could inhibit scientists from writing critical comments (Srivastava, 2014). These examples just cited suggest that when scientists perceive the issues at stake to be important, they are more than willing to participate in public debate. As pointed out in Fox (2014), out of channel reader commentary is relevant mainly for papers that attract extraordinary public attention (“the 1% of scientific papers”). It cannot, therefore, replace more conventional forms of review. Nonetheless, the cases just cited suggest that it can occupy an extremely important niche in science publication.

## Discussion—topics for future research

The results of the survey indicate a sea change in peer review practices, tightly linked to the Internet revolution and to the emergence of Open Access publishing. Twenty years ago almost all channels of scientific publication used some version of classical peer review. Today, scientific publishers offer authors greater choice. The survey identifies two major trends that are already making a major impact, while simultaneously pointing to other potentially significant developments whose immediate impact is smaller.

The first major trend is the rapidly growing role of services that dispense with traditional peer review altogether, allowing authors to publish any article that meets minimum standards for publication and relying on the community to evaluate which of the articles published makes a genuine contribution to science. The well-recognized role of *ArXiv* demonstrates that lack of peer review does not automatically signal poor quality.

The second trend is the rapid growth in non-selective review, as practiced by *PLOS ONE, Frontiers* and many other Open Access publishers. While not quite so rapid as review-less publishing, non-selective review guarantees high acceptance rates, virtually eliminates the delays caused when authors are forced to resubmit articles to multiple journals in sequence and provides fewer opportunities for reviewer bias than traditional review processes. It also provides a stronger guarantee of scientific quality than review-less publication, contributes to the quality of papers, and gives authors the credit associated only with peer-reviewed publications. The *BMC series, Frontiers, PLOS ONE*, and other so-called “mega-journals” or “Series” are growing very rapidly, earning an ever-increasing share of the scientific publishing market. Like review-less publishing, it is clear that there is strong demand for their services.

Alongside these major trends, the survey also identifies several other potentially important developments. One of the most important is the spread of various forms of open review, which remove reviewer anonymity, in many cases publishing review reports alongside the articles to which they refer. Another is the use of various forms of interactive review, in which editors, reviewers and authors work together to improve the quality of a paper. Yet another is the emergence of formal post-publication review, and the increasing importance of informal, out of channel reader commentary, especially critical commentary targeting high profile papers. For the moment, these innovative forms of review and commentary are restricted to a small number of channels. It is likely, however, that the most successful will be imitated by others, becoming more important than they are today.

Of course, none of this implies, that classical peer review is obsolete or that it has lost its role as the “linchpin” of scientific publishing (Sen, 2012). In fact, it is still the preferred review mechanism for the majority of channels in the study. In many cases, the only innovation with respect to review 20 years ago is the introduction of channels for reader commentary, which readers rarely use.

One of the strongest findings of the survey is the persistence of enormous differences between the peer review processes used by different disciplines. In physics, mathematics, and astronomy the use of preprint servers is now standard practice. In the life sciences, the social sciences, the humanities and the arts, it is extremely rare. Despite some signs of change, nearly all channels of publication in the social sciences, humanities and arts use double blind review. In the natural sciences, it is the exception. In the life sciences, mega-journals have given an important role to non-selective review. Outside these disciplines, it is almost unheard of.

Different processes of scientific publication respond to the real or perceived needs of different stakeholders. The interests of well-known authors in well-known institutions are different from those of more junior scholars; mathematicians seeking to verify a proof need different services than natural scientists who want to establish priority for a new result; the needs of readers trying to keep up with developments outside their field are different from those of experimentalists looking for the latest data and protocols in their own specialty. Researchers in different fields bring with them different values and different practices. Given that none of these differences are likely to disappear in the foreseeable future, the most likely scenario for the coming years is continued diversification, with different review procedures serving different author, reader and publisher needs.

The survey presented here has many limitations. In particular, the methods used were designed to capture the broadest possible range of review processes, regardless of their quantitative importance. As a result, it is not a statistically representative sample of the scientific publishing ecosphere. The other main limitations concern the *impact* of the innovations the study describes. Wherever possible, we have cited experimental or observational studies. However, there are relatively few of them, many are more than 10 years old and most refer only to a specific journal or a specific academic discipline. As a result, the survey identified many opinions and hypotheses, but relatively few well-established findings.

Many important claims by innovators have yet to be tested. What are the effects of new review processes on the speed with which articles are published, and on their content and quality? Have they reduced bias against particular categories of author, making scientific publishing more accessible? Have they made it easier to publish replication studies, negative results and hypotheses going against mainstream opinion? Are they more or less effective than traditional practices is detecting scientific malpractice? What has been their effect on the quality of papers accepted for publication? And what has been the effect on readers? Have new publishing practices made it easier or harder for readers to keep up with the latest developments in their own and related disciplines? These are important open questions for future quantitative research.

## Author contributions

RW conceived the study. RW and PS both contributed to data acquisition and analysis, and to the drafting and final approval of the manuscript. Both authors have agreed to be accountable for all aspects of the work related to its accuracy and integrity.

### Conflict of interest statement

Richard Walker is a part-time employee of Frontiers. Pascal Rocha da Silva is a full-time employee of Frontiers.
