# Supplementary material for: Emerging trends in peer review—a survey
Source: Front Neurosci. 2015 May 27;9:169. doi: 10.3389/fnins.2015.00169 (PMC4444765; doi:10.3389/fnins.2015.00169)
Supplement: Supplementary file 1 [file Table1.DOCX]

| **Term** | **Meaning** |
| --- | --- |
| Access review | A review that verifies that a paper meets minimum standards for scientific publication and/or that verifies the credentials of the authors. In some cases (e.g. ArXiv), this is the only review of the paper. |
| Developmental editing | Synonym for interactive review. Generally used to mean collaboration between authors and a professional editor to improve the quality of a paper. In the context of this study, collaboration between authors and reviewers to improve the quality of a text - often online |
| Double Blind review | A review in which authors’ names are not revealed to reviewers and reviewers names are not revealed to authors |
| Endorsement organization | An organization that endorses a paper on a third party repository or journal |
| E-print server | Synonym for preprint server |
| Evaluation | Used by Frontiers to signify the process whereby the community assesses the importance of a scientific publication. |
| Impact Neutral Review | A review process that does not attempt to evaluate the importance or novelty of a paper, but only the soundness of the underlying science. Synonym for non-selective review |
| Interactive review | Synonym for developmental editing. An online review process in which reviewers and editors collaborate to improve the quality of a text |
| Non-selective review | A review process that does not attempt to evaluate the importance or novelty of a paper, but only the soundness of the underlying science. Synonym for impact neutral review |
| Open peer commentary | A review process, based on a formal review procedure, in which authors are invited to submit commentary on a “target” paper, and sometimes, to comment on comments from other reviewers. The commentary is published alongside the paper |
| Open review | A review process, in which reviewers’ identities are revealed to authors. In many cases, reviewers names are published alongside the article they have reviewed, sometimes together with their reports and the complete exchange with authors. In some cases, reviewers decide whether or not their identity should be revealed. |
| Overlay journal | A journal that provides reviews of papers in other publications |
| Portable review | A review suitable for use by multiple journals or which is passed on to other journals after a paper has been rejected by the first journal to which it was submitted. Portable review may take place before submission of a paper. Alternatively, reviews of a paper rejected by one journal may be made available to other journals. In some, but not all cases, the material made available may include the identities of the reviewers |
| Post-Publication Peer Review (PPPR) | A review that occurs only after the paper has already been published online. |
| Pre-print server | A repository that hosts pre-prints of papers that may be subsequently submitted for subsequent publication in journals adopting a conventional peer review process |
| Rebound review | A review process that allows authors whose paper has been rejected in conventional peer review to request an open review by experts proposed by the author ([Sen, 2012](#_ENREF_95); [Ryter and Choi, 2013](#_ENREF_87)) |
| Repository Journal | Synonym for an Overlay Journal: “an open access journal that takes submissions from the preprints deposited at an archive… and subjects them to peer-review”. |
| Simple blind review | A review in which reviewers are anonymous but authors' identity is known to reviewers ([Sen, 2012](#_ENREF_95)) |

Supplementary Materials Table 1: A glossary of search terms used during the review
